# Supplementary material for: Infection-associated gene regulation of L-tartrate metabolism in Salmonella enterica serovar Typhimurium
Source: mBio. 2024 Apr 29;15(6):e00350-24. doi: 10.1128/mbio.00350-24 (PMC11237755; doi:10.1128/mbio.00350-24)
Supplement: Supplemental Figures — Figures S1 to S3. [file mbio.00350-24-s0001.pdf]

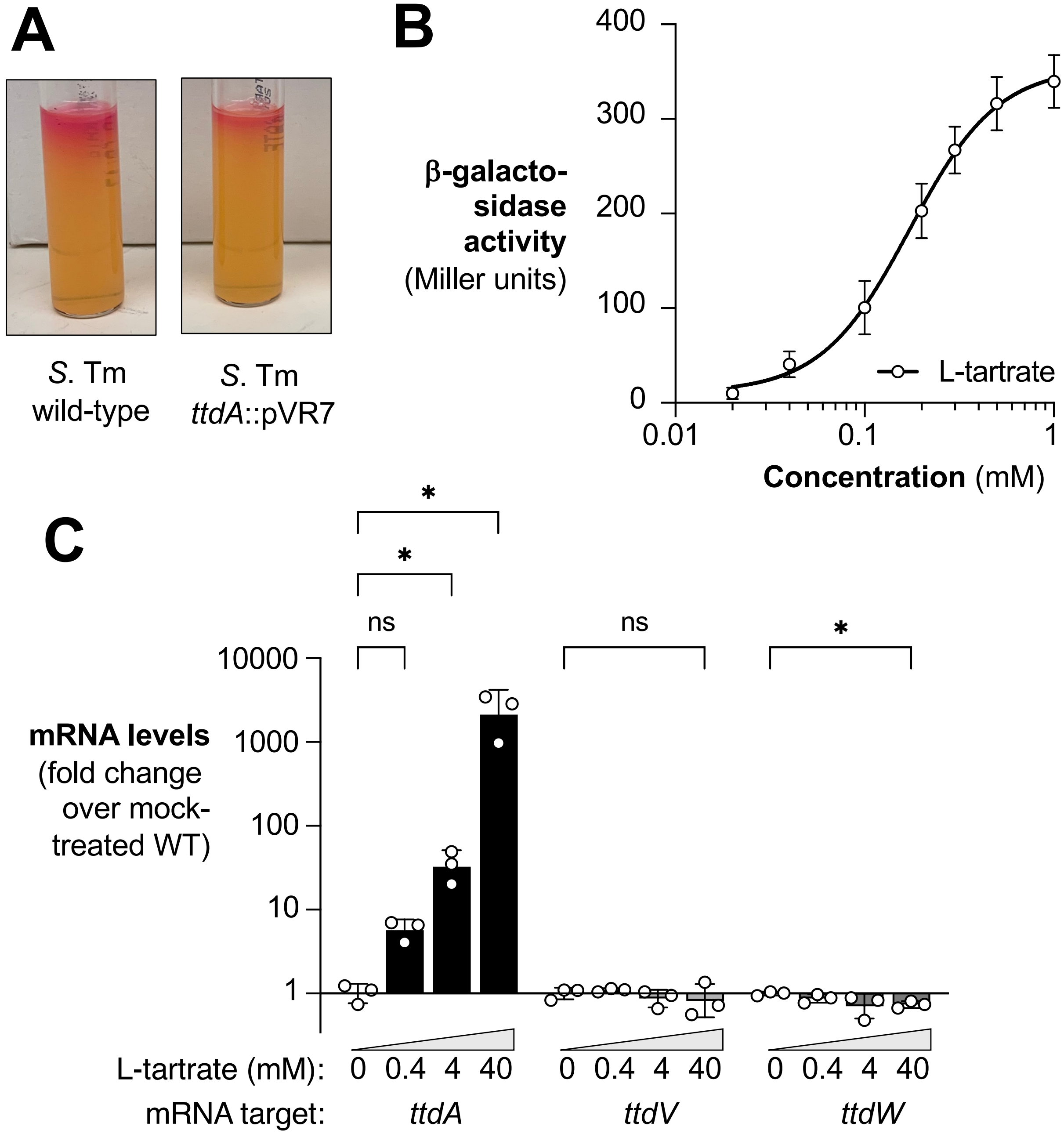

Supplemental Figure S1

(**A**) Jordan's Tartrate agar was inoculated with either the *S.* Tm wild-type strain or the *ttdA-lacZ* transcriptional fusion strain (*ttdA*::pVR7) and incubated overnight at 37 °C. Accumulation of acidic end products from L-tartrate fermentation is indicated by a yellow color.

(**B**) A transcriptional *ttdU-lacZ* fusion strain (*ttdU*::pVR11) was cultured in media containing M9 salts and casamino acids, supplemented with various concentrations of L-tartrate, and grown anaerobically. After 5 hours, β-galactosidase activity was assessed.

(**C**) mRNA levels of *ttdA* (black bars), *ttdV* (light gray bars), and *ttdW* (dark gray bars) were determined by RT-qPCR. The *S.* Tm wild-type strain was cultured in media with M9 salts and casamino acids (M9C), supplemented with increasing concentrations of L-tartrate (0.4 mM, 4 mM and 40 mM) and grown anaerobically for 3 hours. mRNA levels were normalized to the housekeeping gene *gmk*.

Bars represent the geometric mean with geometric standard deviation. Each dot represents one biological replicate.

*, $P < 0.05$; ns, not significant (ANOVA)

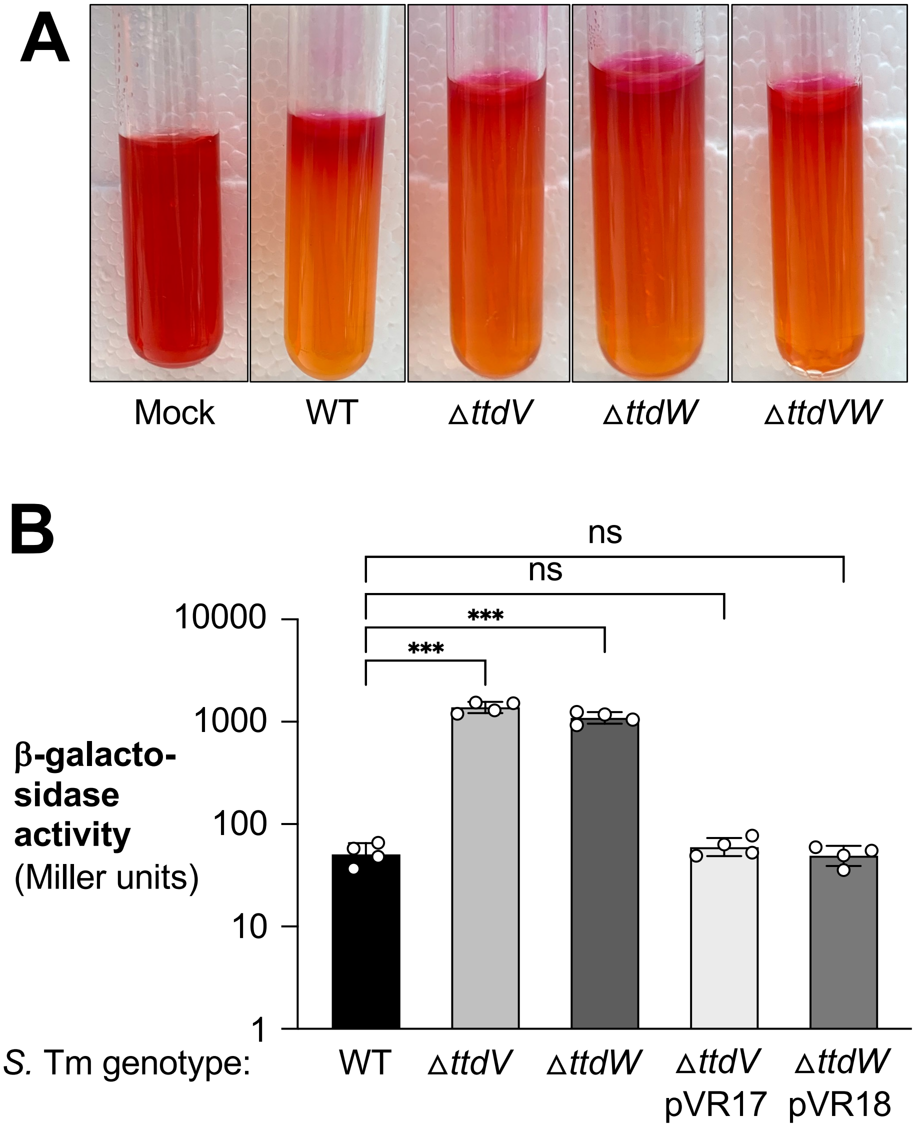

Supplemental Figure S2

(**A**) Jordan's agar base supplemented with L-tartrate was inoculated with the indicated *S.* Tm strains and incubated overnight at 37 °C.

(**B**) The indicated *S*. Tm *ttdA*-*lacZ* transcriptional fusion strains were grown anaerobically without L-tartrate for 3 hours before assessing β-galactosidase activity. The pertinent genotype is listed. For complementation, TtdV-His_6 (pVR17) and TtdW-His_6 (pVR18) were expressed *in trans.*

Bars represent the geometric mean with geometric standard deviation. Each dot represents one biological replicate. WT; wild-type

*** $P < 0.001$; ns, not significant (ANOVA)

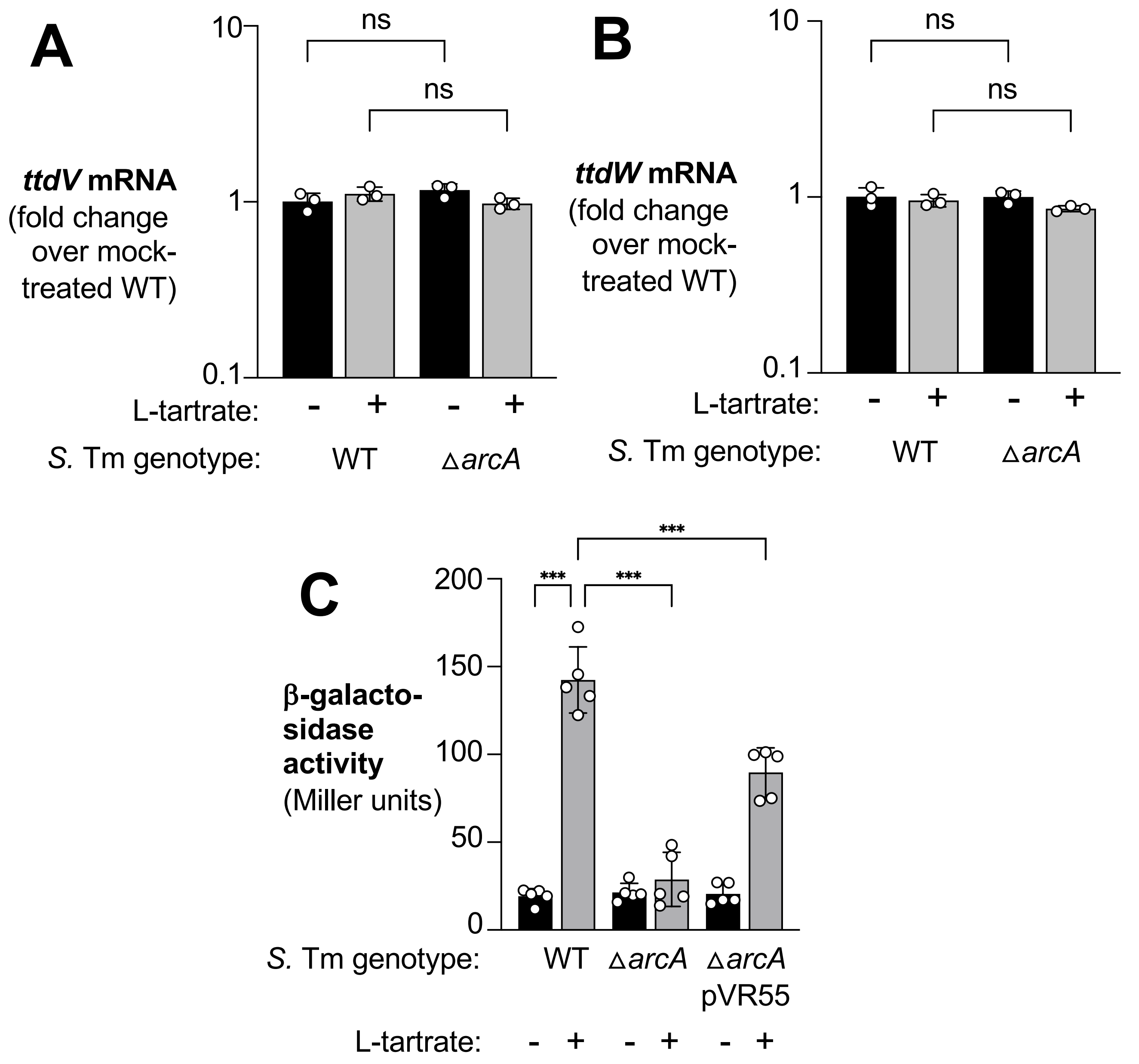

Supplemental Figure S3

(**A and B**) The *S*. Tm wild-type strain and the *arcA* mutant were grown anaerobically in the absence (black bars) or presence (gray bars) of 0.1 mM L-tartrate for 3 hours before quantifying mRNA levels of *ttdV* **(A)** and *ttdW* **(B)** by RT-qPCR.

(**C**) The indicated *S*. Tm *ttdA*-*lacZ* transcriptional fusion strains were grown anaerobically in M9 salts and casamino acids media supplemented without (black bars) or with 0.1 mM L-tartrate (gray bars) for 3 hours before assessing β-galactosidase activity. For complementation, His_6-ArcA was expressed *in trans* (pVR55).

Bars represent the geometric mean with geometric standard deviation. WT; wild-type

*** $P < 0.001$; ns, not significant (ANOVA)
