# Supplementary material for: Infection-associated gene regulation of L-tartrate metabolism in Salmonella enterica serovar Typhimurium
Source: mBio. 2024 Apr 29;15(6):e00350-24. doi: 10.1128/mbio.00350-24 (PMC11237755; doi:10.1128/mbio.00350-24)
Supplement: Supplemental Tables — Tables S1 and S2. [file mbio.00350-24-s0002.docx]

**SUPPLEMENTAL TABLES**

**Table 1:** Recombinant DNA and Bacterial strains used in this study

| **Plasmid** | **Description** | **Reference** |
| --- | --- | --- |
| pET-14b | *ori*(pBR322) T7 promoter N-terminal 6X His Tag Carb^R^ | Novagen/Millipore Sigma |
| pET21(+) | *ori*(pBR322) *ori*(f1) T7 promoter C-terminal 6X His Tag *lacI* Carb^R^ | Novagen/Millipore Sigma |
| pGP705 | *ori*(R6K) *mobRP4* Kan^R^ | (1) |
| pGP706 | *ori*(R6K) *mobRP4 sacRB* Kan^R^ | (2) |
| pFUSE | *ori*(R6K) *mobRP4* Cm^R^ *lacZYA* | (3) |
| pPhoKm | upstream and downstream region of *S.* Tm *phoN* cloned into pGP706 | This study |
| pRDH10 | *ori*(R6K) *mobRP4* Cm^R^ Tet^R^*sacRB* | (4) |
| pMW31 | upstream and downstream region of *S.* Tm *ubiE* cloned into pRDH10 | This study |
| pMW35 | upstream and downstream region of *S.* Tm *menA* cloned into pRDH10 | This study |
| pSC1 | upstream and downstream region of *S*. Tm *ttdV* cloned into pGP706 | This study |
| pSC2 | upstream and downstream region of *S*. Tm *ttdW* cloned into pGP706 | This study |
| pSC3 | Upstream and downstream regions of *S*. Tm *ttdVW* cloned into pGP706 | This study |
| pVR4 | native promoter and gene *ttdW* from *S.* Tm cloned into pPhoKm | This study |
| pVR7 | fragment of *ttdA* in pFUSE | This study |
| pVR8 | native promoter and gene *ttdV* from *S.* Tm cloned into pPhoKm | This study |
| pVR9 | native promoter and genes *ttdVW* from *S.* Tm cloned into pPhoKm | This study |
| pVR11 | Fragment of *ttdU* in pFUSE | This study |
| pVR17 | coding sequence of *S.* Tm *ttdV* cloned into pET21(+) | This study |
| pVR18 | coding sequence of *S.* Tm *ttdW* cloned into pET21(+) | This study |
| pVR55 | coding sequence of *S.* Tm *arcA* cloned into pET14b | This study |
| **Strain** | **Description** | **Reference** |
| DH5α λpir | *E. coli,* F^−^ endA1 hsdR17 (r^−^ m^+^) supE44 thi-1 *recA1 gyrA relA1* Δ(*lacZYA-argF)U189 Φ*80*lacZ*ΔM15 λ*pir* | (5) |
| BL21 (DE3) (pLysE) | *E. coli* B, F *fhuA2 [lon] ompT gal (λ DE3) [dcm] ∆hsdS*; *λ DE3 = λ sBamHIo ∆EcoRI-B int::(lacI::PlacUV5::T7 gene1) i21 ∆nin5* Cm^R^; | (6) |
| S17-1 λpir | *E. coli,* zxx*::RP4 2-(*Tet^R^::Mu*) (*Kan^R^::Tn7*)* λ*pir recA1* *thi pro hsdR* (r^−^ m^+^) | (7) |
| IR715 | *S*. Typhimurium ATCC14028 Nal^R^ | (8) |
| AJB715 | IR715 *phoN*::Kan^R^ | (4) |
| LS24 | IR715 Δ*ttdBA* | (9) |
| LS25 | IR715 Δ*ttdBAU* | (9) |
| MW118 | IR715 Δ*arcB* | (10) |
| MW119 | IR715 Δ*arcA* | (10) |
| MW201 | IR715 Δ*menA* | This study |
| MW208 | IR715 Δ*ubiE* | This study |
| SC1 | IR715 Δ*ttdV* | This study |
| SC2 | IR715 Δ*ttdW* | This study |
| SC3 | IR715 Δ*ttdVW* | This study |
| VR3 | IR715 Δ*ttdW* Δ*phoN*::*ttdW* | This study |
| VR4 | IR715 *ttdA*::pVR7 | This study |
| VR6 | IR715 *ttdU*::pVR11 | This study |
| VR9 | IR715 Δ*ttdV ttdA*::pVR7 | This study |
| VR10 | IR715 Δ*ttdW ttdA*::pVR7 | This study |
| VR11 | IR715 Δ*ttdVW ttdA*::pVR7 | This study |
| VR14 | IR715 Δ*arcA ttdA*::pVR7 | This study |
| VR15 | IR715 Δ*arcB ttdA*::pVR7 | This study |
| VR28 | IR715 Δ*ttdVW* Δ*phoN*::*ttdVW* | This study |
| VR29 | IR715 Δ*ttdV* Δ*phoN*::*ttdV* | This study |
| VR34 | IR715 Δ*ttdV* Δ*phoN*::*ttdV ttdA*::pVR7 | This study |
| VR35 | IR715 Δ*ttdW* Δ*phoN*::*ttdW ttdA*::pVR7 | This study |
| VR36 | IR715 Δ*ttdVW* Δ*phoN*::*ttdVW ttdA*::pVR7 | This study |
| VR48 | IR715 Δ*ubiE ttdA*::pVR7 | This study |
| VR50 | IR715 Δ*menA ttdA*::pVR7 | This study |

**Table 2:** Primers used in this study.

| **Targeted mutagenesis and complementation constructs** | | |
| --- | --- | --- |
| Purpose | Sequence | Reference |
| Construction of plasmid pPhoKm | 5’-AAGCTTCTTCTAGAGGTACCAAGGCAAGCCGGTAAAAAC-3’  5’-ACTTTCACCTGCATGCGGATCAGGCAATAATAAACAGATG-3’  5’-TGCCTGATCCGCATGCAGGTGAAAGTCTTCCCGC-3’  5’-TCCCGGGAGAGCTCGATATCGTTCGTTTGGATCAAACACC-3’ | This study |
| Deletion of *menA* in *S*. Tm | 5’-GCCATCTCCTTGCATGCGTCTATGTTCCAGAACCTG-3’  5’-ATTTGTCAGATATTTAGCGCCAATAAAAATGG-3’  5’-CGCTAAATATCTGACAAATATCAATTAACAATTGATG-3’  5’-CAAGGAATGGTGCATGCAGTCACATAGTGGCCTATG-3’ | This study |
| Deletion of *ubiE* in *S*. Tm | 5’-GCCATCTCCTTGCATGCATGTGGTCATCGACGCTAAAATG-3’  5’-CCACCCTCCTGCTTATCGATAAAAATGTTCAAGAAG-3’  5’-GATAAGCAGGAGGGTGGAATGCCTTTTAAAC-3’  5’-CAAGGAATGGTGCATGCATCTTTATGCCGGTTTGG-3’ | This study |
| Deletion of *ttdW* in *S*. Tm | 5’-GCTTCTTCTAGAGGTACCGCATGTACAGAGCAACATTTTCATTTTTTC-3’  5’- CATTACGCAAATAATGCGTCAACATATGAAG -3’  5’-GACGCATTATTTGCGTAATGTGATCGCG-3’  5’-GGAGAGCTCGATATCGCATGCTGACTAAACGTATTCAGAACG-3’ | This study |
| Deletion of *ttdV* in *S*. Tm | 5’-gcttcttctagaggtaccgcatgATGACGCCGCCCACTTTG-3’  5’-ttcagcgaaaATGGAGAATATGCTGACCCGC-3’  5’-tattctccatTTTCGCTGAAAGAAGCTTAATC-3’  5’-ggagagctcgatatcgcatgACTCGAACAGGAAGGTTTTTTAATC-3’ | This study |
| Deletion of *ttdVW* in *S*. Tm | 5’-gcttcttctagaggtaccgcatgATGACGCCGCCCACTTTG-3’  5’-cattacgcaaATGGAGAATATGCTGACCCGC-3’  5’-tattctccatTTGCGTAATGTGATCGCG-3’  5’-ggagagctcgatatcgcatgCTGACTAAACGTATTCAGAACG-3’ | This study |
| Complementation of *ttdW* in *S*. Tm | 5’-TTTATTATTGCCTGATCCGCATGTGTGGAAGGCGACGGTCAG-3’  5’-GGAAGACTTTCACCTGCATGTTAGGCTTCTGCACTTTCCCAATC-3’ | This study |
| Complementation of *ttdV* in *S*. Tm | 5’-TTTATTATTGCCTGATCCGCATGTGATACGTGCGCAAGCGC-3’  5’-GGAAGACTTTCACCTGCATGTCAGGCAGAAGGGTCACC-3’ | This study |
| Complementation of *ttdVW* in *S*. Tm | 5’-TTTATTATTGCCTGATCCGCATGTGTGGAAGGCGACGGTCAG-3’  5’-GGAAGACTTTCACCTGCATGTCAGGCAGAAGGGTCACC-3’ | This study |
| **qPCR target sequences** | | |
| *ttdW* | 5’–GGCTGTCGCAGAAGACAAAT-3’  5’–CCGTCCAGGAACTGCTGATA-3’ | This study |
| *ttdV* | 5’–TGGTGTTGGGCATCAATGAA-3’  5’–CCACCGCCTCACTTTCTAAC-3’ | This study |
| *ttdU* | 5’–CGTTCATGTTGCTGATCG-3’  5’–GTCGTTGCGGTATTGGAC-3’ | This study |
| *ttdA* | 5’–GTGCAGGCTATGAAGGTGTG-3’  5’–ACGCTCATTCTCGTTATGCG-3’ | This study |
| *ttdB* | 5’–GGGTAAAGGCGGTATGGGTA-3’  5’–ATTTCTTCCACGCACACGG-3’ | This study |
| **EMSA probe sequences** | | |
| Probe 1 | 5’-[Biotin]CCATCCGTGAACACTATGAAAT-3’  5’–[Biotin]GCCTTCTTCGGTGACCTTAT-3’ | This study |
| Probe 2 | 5’-[Biotin]CCTAACTCGGCGGAATACTC-3’  5’–[Biotin]CAACATAGCGGGTCAGCAT-3’ | This study |
| Probe 3 | 5’-[Biotin]ACCGAAGAAGAGTATGCCGT-3’  5’–[Biotin]GCTACATCAAGATCGTGGTTAGA-3’ | This study |
| Probe 4 | 5’-[Biotin]CGTATGCTGACCCGCTATG-3’  5’–[Biotin]GGGCGAACACCAATAAACAT-3’ | This study |

**SUPPLEMENTAL REFERENCES**

1. Hughes ER, Winter MG, Duerkop BA, Spiga L, Furtado de Carvalho T, Zhu W, Gillis CC, Buttner L, Smoot MP, Behrendt CL, Cherry S, Santos RL, Hooper LV, Winter SE. 2017. Microbial Respiration and Formate Oxidation as Metabolic Signatures of Inflammation-Associated Dysbiosis. Cell Host Microbe 21:208-219.

2. Gillis CC, Hughes ER, Spiga L, Winter MG, Zhu W, Furtado de Carvalho T, Chanin RB, Behrendt CL, Hooper LV, Santos RL, Winter SE. 2018. Dysbiosis-Associated Change in Host Metabolism Generates Lactate to Support Salmonella Growth. Cell Host Microbe 23:54-64.e6.

3. Bäumler AJ, Tsolis RM, van der Velden AW, Stojiljkovic I, Anic S, Heffron F. 1996. Identification of a new iron regulated locus of Salmonella typhi. Gene 183:207-13.

4. Kingsley RA, Humphries AD, Weening EH, De Zoete MR, Winter S, Papaconstantinopoulou A, Dougan G, Baumler AJ. 2003. Molecular and phenotypic analysis of the CS54 island of Salmonella enterica serotype typhimurium: identification of intestinal colonization and persistence determinants. Infect Immun 71:629-40.

5. Pal D, Venkova-Canova T, Srivastava P, Chattoraj DK. 2005. Multipartite regulation of rctB, the replication initiator gene of Vibrio cholerae chromosome II. J Bacteriol 187:7167-75.

6. Daegelen P, Studier FW, Lenski RE, Cure S, Kim JF. 2009. Tracing ancestors and relatives of Escherichia coli B, and the derivation of B strains REL606 and BL21(DE3). J Mol Biol 394:634-43.

7. Simon R, Priefer U, Pühler A. 1983. A Broad Host Range Mobilization System for In Vivo Genetic Engineering: Transposon Mutagenesis in Gram Negative Bacteria. Bio/Technology 1:784-791.

8. Stojiljkovic I, Baumler AJ, Heffron F. 1995. Ethanolamine utilization in Salmonella typhimurium: nucleotide sequence, protein expression, and mutational analysis of the cchA cchB eutE eutJ eutG eutH gene cluster. J Bacteriol 177:1357-66.

9. Spiga L, Winter MG, Muramatsu MK, Rojas VK, Chanin RB, Zhu W, Hughes ER, Taylor SJ, Faber F, Porwollik S, Carvalho TF, Qin T, Santos RL, Andrews-Polymenis H, McClelland M, Winter SE. 2023. Byproducts of inflammatory radical metabolism provide transient nutrient niches for microbes in the inflamed gut. bioRxiv doi:10.1101/2023.12.08.570695.

10. Gillis CC, Winter MG, Chanin RB, Zhu W, Spiga L, Winter SE. 2019. Host-Derived Metabolites Modulate Transcription of Salmonella Genes Involved in l-Lactate Utilization during Gut Colonization. Infect Immun 87.
